# The Treatment of Pulpless Teeth

**Published:** 1881-12

**Authors:** C. M. Wright

**Affiliations:** Basle Switzerland


### THE TREATMENT OF PULPLESS TEETH.

BY DR. C. M. WRIGHT.

*Basle Switzerland.*

*Read before the American Dental Society of Europe, Aug.* 12, 1881.

Mr. President and Gentlemen:—As I am sensative about the rebuke that might be inflicted upon me if I do not come up to time when called upon, and as I have never failed to put in some sort of an appearance in this ring every time that "*Time*" has been called, I have left the knee of my trainer and staggered into the ring this ninth round in rather a damaged condition. Not as regards my peepers, bread basket, pins, etc., nor that I have lost much claret, but my intellectual joints are getting so stiff from rather a long pause between the rounds that unless some amiable buffer or friendly "vagrant" considerately comes forward and knocks me down, I shall have to throw up the sponge. Your executive committee has appointed me as one of three, to essay something on the "treatment of pulpless teeth and the manner of filling roots." During ten years I have drilled very many small tap holes or vent holes under the margins of the gums into aching molars and bicuspids in the superior and inferior maxillary arches, and have left these with no further treatment. So large a majority of these teeth are doing well as masticators that I am not ashamed to tell you here of this practice.

When teeth have been presented without filling, and when the roots have been pulpless or filled with the putrifying corpses of pulps, I have washed out with tepid water and *very carefully* excavated with barbed "nerve extractors," reamers, etc., fearing that the disturbance may cause active inflammation and pain, especially if the mass in the roots presents that soft, pulpy, and very offensive sort. If the canal is small and the contents dry and dusty, I have not the same fear of inflammation, but can ream more freely and introduce immediately a trial filling of carbolized cotton in the canal and red gutta percha or wax in the crown cavity. If this causes no pain in a day or two, I do not hesitate to fill with anything that I may fancy at the moment, and I have records of lead (commercial) cut into plugs with a knife and forced into large roots—of common tin foil containing lead and other impurities—of gold foil—of carbolized cotton and red gutta percha—of oxy-chlorid of zinc blown and pumped into the canal—of fiddle strings dipped in creosote—of cotton and sandarac—of cotton and gum copal in ether, etc.

In obstinate cases when the patient gets a swelled face every time he looks through a key-hole, and where careful handling—trial fillings—creosote — alcohol — warm water injections—permanganate of potassa injection—swabbings of aconite and iodine

—private internal swearings with a severe and patient face—in fact, where from one cause or another well known to you the case is obstinate, and I can't go in and reconstruct the whole physical character of the patient, and can't clear out the influence of several generations of corruption and can't get the patient to help in any way to try to clear out the "cock-eyed browsers on the dental territory" and the bacteria that feed on the corruption ever present—just where some transplant, I clean out as well as I can and leave a door in the shape of a vent under the gums where the animals can go in and out at pleasure, lay a floor over the passage, of platina or gutta percha and fill the crown. These teeth do half service for a long time and are no worse perhaps for the patient than six cups of coffee at a meal—twenty cigars a day, encisted bullets in the legs, lobster salad at mid-night, and perhaps an unwashed epidermis. We are not on the subject of alveolar abscess and its treatment, or chronic peridontitis—therefore I have tried to confine myself to the consideration of the mechanical treatment of pulpless teeth.

In conclusion let me say that I am certain we save thousands of teeth to-day that formerly were condemned without a trial to the lynch process of the forceps or key, and while as in medical practice we lose patients, or teeth, occasionally, our patient *trying*, and more careful manipulation is having its good results in saving teeth, and it strikes me that this is what we are dentists for.

